# MHC class I-related chain A and B ligands are differentially expressed in human cervical cancer cell lines

**DOI:** 10.1186/1475-2867-11-15

**Published:** 2011-06-01

**Authors:** Susana del Toro-Arreola, Naela Arreygue-Garcia, Adriana Aguilar-Lemarroy, Angel Cid-Arregui, Miriam Jimenez-Perez, Jesse Haramati, Patricio Barros-Nuñez, Oscar Gonzalez-Ramella, Alicia del Toro-Arreola, Pablo Ortiz-Lazareno, Georgina Hernandez-Flores, Alejandro Bravo-Cuellar, Adrian Daneri-Navarro, Luis F Jave-Suarez

**Affiliations:** 1 Laboratorio de Inmunología, Departamento de Fisiología, Centro Universitario de Ciencias de la Salud, Universidad de Guadalajara, Guadalajara, Jalisco, México; 2 División de Inmunología, Centro de Investigación Biomédica de Occidente, Instituto Mexicano del Seguro Social, Guadalajara, Jalisco, México; 3 Translational Immunology Unit, German Cancer Research Center (DKFZ) Im Neuenheimer Feld 280 69120-Heidelberg, Germany; 4 División de Genética, Centro de Investigación Biomédica de Occidente, Instituto Mexicano del Seguro Social, Guadalajara, Jalisco, México

**Keywords:** MICA, MICB, Cervical cancer

## Abstract

**Background:**

Natural killer (NK) cells are an important resource of the innate immune system directly involved in the spontaneous recognition and lysis of virus-infected and tumor cells. An exquisite balance of inhibitory and activating receptors tightly controls the NK cell activity. At present, one of the best-characterized activating receptors is NKG2D, which promotes the NK-mediated lysis of target cells by binding to a family of cell surface ligands encoded by the MHC class I chain-related (MIC) genes, among others. The goal of this study was to describe the expression pattern of MICA and MICB at the molecular and cellular levels in human cervical cancer cell lines infected or not with human papillomavirus, as well as in a non-tumorigenic keratinocyte cell line.

**Results:**

Here we show that MICA and MICB exhibit differential expression patterns among HPV-infected (SiHa and HeLa) and non-infected cell lines (C33-A, a tumor cell line, and HaCaT, an immortalized keratinocyte cell line). Cell surface expression of MICA was higher than cell surface expression of MICB in the HPV-positive cell lines; in contrast, HPV-negative cells expressed lower levels of MICA. Interestingly, the MICA levels observed in C33-A cells were overcome by significantly higher MICB expression. Also, all cell lines released higher amounts of soluble MICB than of soluble MICA into the cell culture supernatant, although this was most pronounced in C33-A cells. Additionally, Real-Time PCR analysis demonstrated that MICA was strongly upregulated after genotoxic stress.

**Conclusions:**

This study provides evidence that even when MICA and MICB share a high degree of homology at both genomic and protein levels, differential regulation of their expression and cell surface appearance might be occurring in cervical cancer-derived cells.

## Background

The immune system endows vertebrates with the ability to detect and respond to various challenges to the homeostasis of the organism; among these, is the emergence of nascent tumors. NK cells are major innate effectors for the early recognition of transformed cells because they can spontaneously detect their targets without prior sensitization [1]. The killing activity is mainly controlled by an exquisite balance of competing inhibitory and activating receptors [2,3]. One of the best characterized activating receptors is NKG2D, a C-type lectin-like activating immunoreceptor whose expression is confined to NK cells, CD8^+ ^T cells and γδ T cells [4,5]. More recently, the expression of NKG2D has been described also in a small population of CD4^+^CD28^- ^T cells in patients with autoimmune conditions [6]. In humans, NKG2D recognizes two structurally distinct families of ligands named MHC class I chain-related (MIC) molecules, and the UL16-binding proteins (ULBPs) 1 to 5, originally identified through interactions with the cytomegalovirus UL16 glycoprotein. Both MIC and ULBP proteins engage NKG2D, which then triggers the cytokine production and cytotoxic activity seen in activated NK cells [7-11].

It has been reported that MICA and MICB are weakly expressed on healthy cells [12-14], but they can be up-regulated by actively growing epithelial and hematological tumors [15-20]. The molecular mechanisms involved in the expression of MICA and MICB are still poorly understood; however, experimental evidence has revealed that their over-expression is the result of a DNA damage response that involves the ATM (ataxia telangiectasia mutated) and the ATR (ATM- and Rad3-related) protein kinases [21,22]. Interestingly, it has been demonstrated that the ATM/ATR pathway is crucial for the regulation of the nuclear factor NF-κB after the induction of genotoxic stress [23] and NF-κB has been shown to be involved in the expression of MICA in T cells [24]. Despite the fact that MICA and MICB share a high homology at the DNA and protein level, there is evidence for differential regulation of their promoters [25] indicating that these molecules could respond dissimilarly to several damage stimuli.

On the other hand, cervical cancer is one of the most common malignant tumors in women worldwide, [26,27]. Clinical, molecular and epidemiological data have identified infection with human papillomavirus (HPV) as a necessary cause for the development of this tumor [28-31]. To date, over 100 different types of HPV have been identified, and approximately 13 are considered as high-risk types with capability to transform cells in the genital tract, with types 16, 18, 31, and 45 being the most predominant types of HPV associated with high-grade intraepithelial lesions and invasive cancer [32-34]

This study was focused on gaining a better understanding of MICA and MICB expression at the molecular and cellular levels in human cervical cancer cell lines infected or not with HPV and a non-tumorigenic keratinocyte cell line. Our data provides evidence that despite sharing a high degree of homology, MICA and MICB might be differentially regulated at the transcriptional and protein level. Therefore, it will be necessary to elucidate if MICA and MICB acting together or individually are important participants in NKG2D-mediated activation pathways in patients with HPV- associated tumors.

## Materials and methods

### Cell lines and cell cultures

The human cervical cancer-derived cell lines (HeLa, SiHa and C33-A) and the spontaneously immortalized human epithelial cell line HaCaT (kindly obtained from Dr. Boukamp, DKFZ-Heidelberg, Germany), which lacks tumorigenic properties [35] were maintained in Dulbecco's modified Eagle's medium containing glutamax, 10% fetal bovine serum, penicillin (100 U/mL) and streptomycin (100 μg/mL). The aforementioned products were obtained from GIBCO™ Invitrogen Corporation, Carlsbad, CA, USA. Cultures were maintained at 37° C in a humidified atmosphere with 5% CO_2_.

### Etoposide treatment

Cultures of HaCaT and HeLa cells were treated with etoposide (Laboratorios Lemery S.A. de C.V., México, D.F.) at a concentration of 170 μM. Cells were incubated at 37° C for 4 hours and used for RNA extraction.

### MICA and MICB cell surface expression by flow cytometry

Surface MICA and MICB expression was evaluated by flow cytometry. Briefly, cells from the different cultures (HeLa, SiHa, C33-A and HaCaT) were harvested by scraping and washed with PBS. Cells were adjusted at 8 × 10^5 ^cells/mL and incubated with primary mAb for 45 min at 4°C in the dark. Isotype controls were also included. Finally, cells were analyzed using FACS Aria (BD Biosciences). The following mouse mAbs were used: PE-conjugated anti-MICA/B (clone 6D4, catalog number sc-23868, Santa Cruz Biotech, Santa Cruz, CA, USA), APC-conjugated anti-hMICA (clone 159227, catalog number FAB1300A, R & D Systems, Inc. Minneapolis, MN, USA) and APC-conjugated anti-hMICB (clone 236511), catalog number FAB1599A, R & D Systems, Inc. Minneapolis, MN, USA).

### SDS-PAGE and Western blot analysis of MICA/B

Cells were harvested by scraping and then lysed with RIPA buffer by sonication (10 pulses, 90% amp). Extracts were incubated for 40 min at 4°C and obtained by centrifugation (14000 rpm for 5 min at 4°C). Protein concentration was determined using a protein quantitation kit (DC Protein Kit, BioRad, Hercules, CA, USA) and 40 μg of whole-cell extract from each cell type were analyzed by electrophoresis using a 12.5% SDS-PAGE. Proteins were then transferred to a PVDF membrane (Millipore Corporation, Bedford, MA, USA) and incubated with 5% w/v nonfat dry milk to block nonspecific binding. Primary antibody anti-MICA/B (clone F-6, Santa Cruz Biotech, Santa Cruz, CA, USA) was incubated overnight at 4°C and secondary antibody was incubated with the membrane for 1 h at RT, followed by chemiluminiscent detection (Millipore Corporation, Bedford, MA, USA) using Kodak BioMax Light films. MICA/B were visualized by the presence of a band of approximately 62 kDa. The images were obtained by computerized scanning of films.

### Transcriptional expression of MICA and MICB by Real-Time RT-PCR

Total RNA was isolated from cells with the PureLink™ Micro-to-Midi Total RNA Purification System (Invitrogen Corp., Carlsbad, CA, USA), as described by the manufacturer. cDNA synthesis was performed using 5 μg of total RNA primed with oligo(dT) using the SuperScript™ III First-Strand Synthesis System for RT-PCR (Invitrogen Corp., Carlsbad, CA, USA). Real time PCR analysis was performed using the LightCycler-FastStart DNA Master^PLUS ^SYBR Green I Kit (Roche Applied Science, Mannheim, Germany) using the LightCycler 1.5 System (Roche, Mannheim, Germany). In each assay, standard curves with four serial dilution points of the cDNA mixture and a negative control were included. The relative quantification analysis for gene expression was performed with the LightCycler Software v4 using GAPDH, β-Actin and L32 ribosomal protein as reference genes. The oligonucleotides for MICA, MICB and constitutive genes are described in Table 1.

**Table 1:** Oligonucleotides used in Real-Time RT-PCR assays

Gene	Oligonucleotides (5'- 3')
*MICA F*	CAG ACT GCC TGC AGG AAC TA
*MICA R*	TTT CTT CTT ACA ACA ACG GAC ATA
*MICB F*	CGG ACA GAC TTT CCA TAT GTT T
*MICB R*	TCC AAC AAC AAT AAA TAA GTG ATG
*β-Actin F*	TCC GCA AAG ACC TGT ACG
*β-Actin R*	AAG AAA GGG TGT AAC GCA ACT A
*GAPDH F*	CAC TGC CAC CCA GAA GAC TGT G
*GAPDH R*	TGT AGG CCA TGA GGT CCA CCA C
*RPL32 F*	GCA TTG ACA ACA GGG TTC GTA G
*RPL32 R*	ATT TAA ACA GAA AAC GTG CAC A

### Quantification of soluble MICA and soluble MICB in cell culture supernatants by ELISA assay

MICA and MICB ELISA kits (catalog numbers DY1300 and DY1599, respectively both from R&D Systems, Inc. Minneapolis, MN, USA) were used to detect soluble MICA and soluble MICB in cell culture supernatants (depleted of <10 kDa proteins) of HaCaT, C33-A, SiHa and HeLa cell cultures, following the manufacturer's protocol. Absorbance values (at A_450_) by duplicate were plotted against dilutions and expressed as pg/mL.

### Ethical approval

This Protocol was approved by the Committees of Research, Ethics & Biosafety with the registration number CI-4608 of the Centro Universitario de Ciencias de la Salud - Universidad de Guadalajara and by the Ethical Board CLIS-1305 of the Centro de Investigación Biomédica de Occidente - IMSS with the registration number R-2007-1305-1.

## Results

### Cell surface expression of MICA/B in cervical cancer-derived cell lines and non-tumorigenic keratinocytes

**Figure 1:**
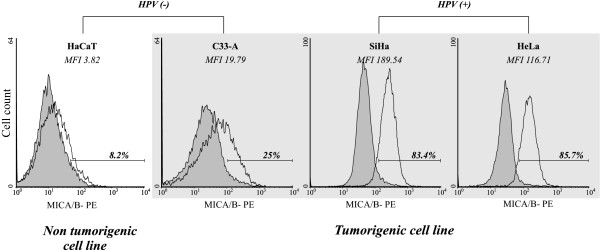
**MICA/B is differentially expressed in cervical cancer-derived cell lines**. The cell surface expression of MICA/B was detected by flow cytometry using an antibody that recognizes both MICA and MICB (open curves); IgG2a antibody isotype-control was also included (filled curves). The histograms show the mean fluorescence intensity (MFI) and the percentage of MICA/B-expressing cells. Results were analyzed using the program WinMDI.

There is evidence that overexpression of MICA/B ligands, combined with other factors, is associated with the survival and prognosis of cervical cancer patients [36]. However, the role that HPV infection plays in the regulation of MICA/B ligand expression still remains unclear. In order to elucidate this point, we addressed cell surface expression of MICA/B in various established cervical tumor cell lines known to be infected with HPV-18 (HeLa), HPV-16 (SiHa) or uninfected (C33-A). Additionally, we included a human non-tumorigenic immortalized cell line derived from epidermal keratinocytes (HaCaT), which has maintained the classical morphological characteristics of differentiation comparable with those of normal keratinocytes [35]. To assess simultaneously the expression of MICA and MICB we used a mAb that recognizes both molecules (clone 6D4). As shown in Figure 1, a high percentage of MICA/B-expressing cells with strong staining were observed in the HPV infected SiHa and HeLa cell lines (83.4%/MFI 189.54 and 85.7%/MFI 116.71, respectively). Conversely, only a low percentage of HPV-negative C33-A cells and the non-tumorigenic HaCaT cells were weakly positive for surface expression of MICA/B (25%/MFI 19.79 and 8.2%/MFI 3.82, respectively). Due to the fact that we did not assess the expression of MICA/B ligands in primary HPV-infected keratinocytes, we can not discard the possibility that HPV infection might be influencing the regulation of MICA/B cell surface expression.

### MICA/B expression cell lysates from cervical cancer-derived cell lines and non-tumorigenic keratinocytes

**Figure 2:**
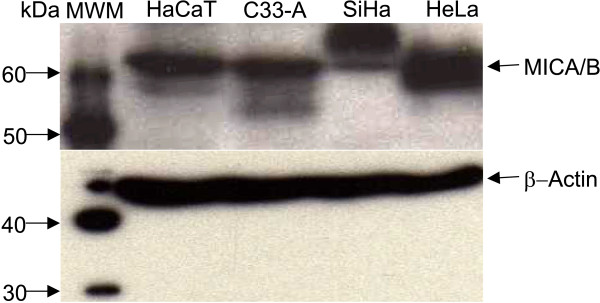
**MICA/B expression in total protein extracts from cervical cancer-derived cell lines and non-tumorigenic keratinocytes**. Whole lysates from HaCaT, C33-A, SiHa and HeLa cells were electrophoresed in SDS-PAGE gels under reductive conditions and subjected to Western blot. A compact band of approximately 62 kDa was visualized in almost all the cell lines. SiHa instead revealed a band heavier than 62 kDa (β-Actin was used as loading control).

To determine whether the observed differences in cell surface expression of MICA/B between HPV-positive and -negative cell lines could be attributed to differences in protein expression, we assessed their expression in cell lysates by Western blot using a mAb. As shown in Figure 2, in all cell lines this antibody recognized a band of approximately 62 kDa, which corresponds with the predicted MICA/B molecular weight. The analysis of the signal of MICA/B in each cell line, after normalizing for β-Actin, revealed that despite the lower surface expression observed on HaCaT and C33-A cells by flow cytometry, these uninfected cells actually expressed comparable amounts of total MICA/B protein. Interestingly, while SiHa cells revealed a predominant band heavier than 62 kDa, which could correspond to a higly glycosylated form of MICA/B, C33-A cells showed a lighter product, which could actually correspond to MICB, due to this molecule has five possible *N*-linked-glycosylation sites (MICA has eight glycosylation sites). However, we did not perform further experiments to verify this finding.

### Cell surface and soluble MICA and MICB expression

**Figure 3:**
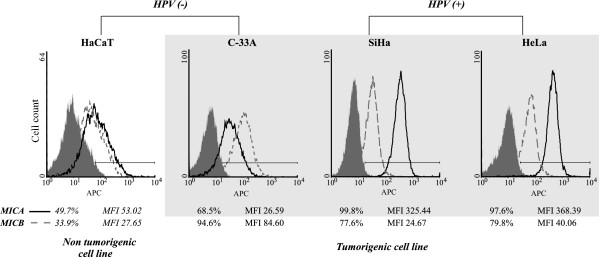
**Cell surface MICA and MICB expression in cervical cancer-derived cell lines and non-tumorigenic keratinocytes**. Flow cytometry analysis to detect MICA and MICB separately was carried out on HaCaT, C33-A, SiHa and HeLa cells to determine the percentage and the mean fluorescence intensity of MICA and MICB. Results were analyzed using the program WinMDI (filled gray curves: isotype control, black curves: anti-MICA, gray curves: anti-MICB).

Next, we repeated our measurements of MICA/B on the cell surface, this time using specific anti-MICA and anti-MICB mAbs, which do not cross-react with each other. Additionally, soluble MICA and MICB released into the cell culture supernatants were also quantified. As shown in Figure 3, nearly 100% of SiHa (HPV-16) and HeLa (HPV-18) cells exhibited cell surface expression of MICA with median fluorescence intensities (MFI) of 325.44 and 368.39, respectively. In contrast, the HPV-negative cell lines C33-A and HaCaT cells showed significantly lower values of MICA expression with 68.5% (MFI 26.59) and 49.7% (MFI 53.02) of positive cells, respectively. For MICB, a higher percentage of positive cells was detected in C33-A cells (94.6% MFI 84.6) than in HeLa (79.8%, MFI 40.06), SiHa (77.6%, MFI 24.67) and HaCaT cells (33.9%, MFI 27.65). Interestingly, the intensity of staining observed in C33-A cells using the specific anti-MICB mAb was much stronger than that observed with the antibody recognizing MICA/B (clone 6D4), suggesting that the later antibody might have a lower affinity for MICB. In summary, these results show a significant expression of MICA molecules on the cell surface of HPV-positive cell lines compared to the HPV-negative cells. Conversely, our data demonstrate that cell surface expression of MICB was present in all the cell lines independently of the HPV presence.

Interestingly, there is a discrepancy between the FACS and Western blot experiments. We in fact expected to find differences in the expression of MICA/B detected by FACS and Western blot, since there are mechanisms of shedding that regulate the availability of these ligands at the cell surface. Additionally, there is evidence that shedding (proteolytic) mechanisms can be modulated by the presence of HPV. Thus, due to the observed shedding of MICA and MICB from the cell surface, we expected a certain amount of the total MIC signal observed by Western blot will be "lost" to cell surface FACS analysis.

**Figure 4:**
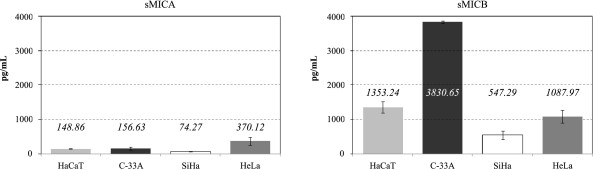
**Soluble MICA and MICB in supernatants from human cervical cancer cell lines and non-tumorigenic keratinocytes**. Levels of soluble MICA and soluble MICB were measured in concentrated supernatants of HaCaT, C33-A, SiHa and HeLa cells after three days of cell culture by ELISA assays using the corresponding anti-MICA or anti-MICB monoclonal antibodies. Absorbance values (at A_450_) measured by duplicate were plotted against standard dilutions and were expressed as pg/mL.

Next, the release of soluble MICA and MICB into the cell culture supernatants of subconfluent cultures of HaCaT, C33-A, SiHa and HeLa cells was analyzed by ELISA. As shown in Figure 4, all four-cell lines released more soluble MICB than soluble MICA, and this effect was more pronounced in C33-A cells, which exhibited titers as high as 3830.65 pg/mL. This finding was similar to the result obtained by flow cytometry, which had revealed a high cell surface expression of MICB in C33-A cells. On the other hand, the highest level of soluble MICA was found in supernatant of HeLa cells, reaching 370.12 pg/mL. Taken together, these results point to a preferential release of MICB by the four cell lines, with the highest levels in HPV-negative cell lines.

### Quantitative analysis of MICA and MICB mRNA

**Figure 5:**
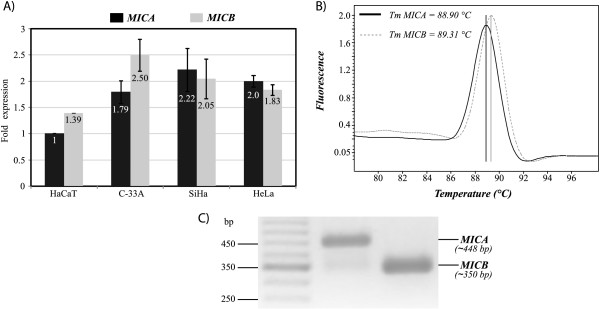
**Relative quantification analysis of MICA and MICB genes**. A) Relative expression of MICA and MICB in each cell line was performed by Real-Time PCR using GAPDH, β-Actin and L32 ribosomal protein as reference genes. The "fold expression" (ordinates) was obtained by normalizing the data values taking MICA expression of HaCaT cells as 1. The figure represents the average ± SD of the relative expression obtained with the reference genes. B) Temperature melting analysis performed by Real-time RT-PCR shows amplification of only one specific peak for each set of primers. C) Specificity of MICA and MICB amplification fragments was demonstrated by running the amplification products on 1.5% agarose gel, giving a fragment of approximately 448 bp for MICA and 350 bp for MICB.

To address the possibility that the differences observed in cell surface expression of MICA and MICB between cell lines could be due to a different amount of mRNA, we performed quantitative analysis of MICA and MICB mRNAs by Real-Time RT-PCR using GAPDH, β-Actin and the ribosomal L32 transcripts as reference genes. MICA and MICB mRNA in HeLa, SiHa and C33-A cells were normalized against those obtained with HaCaT cells, since these cells were considered as non-transformed, non-tumorigenic cells. As shown in Figure 5, C33-A, SiHa and HeLa cells exhibited higher relative expression of MICA (1.79, 2.22 and 2, respectively) when compared with MICA mRNA levels of HaCaT cells (whose value was normalized to 1). For MICB, we observed in C33-A cells (2.5 fold increase over the HaCaT MICA standard), followed by SiHa (2.05 fold), HeLa (1.83 fold) and HaCaT (1.39 fold). In contrast with the other three cell lines, MICB mRNA abundance was higher than MICA mRNA abundance in C33-A (Figure 5). These data are consistent with the data obtained by flow cytometry, where we observed a higher expression of MICB than MICA (see Figure 3). Specific amplification of MICA (amplicon of 448 bp) and MICB (amplicon of 350 bp) were observed in 2% agarose gels (Figure 5 shows the results obtained with HeLa cells).

### Genotoxic stress induces preferential MICA expression

**Figure 6:**
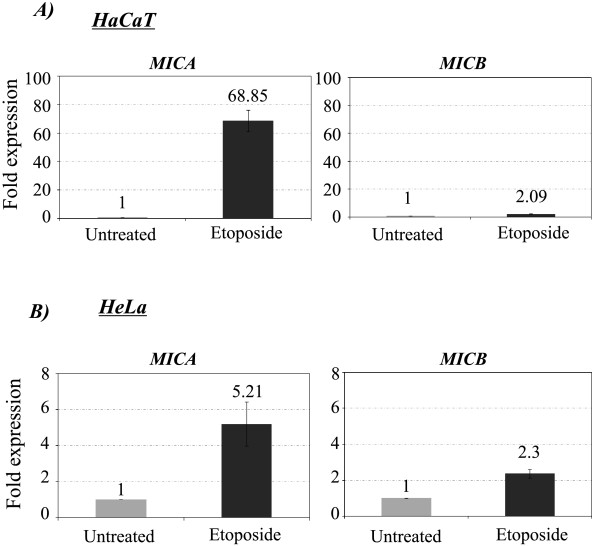
**Relative quantification analysis of the expression levels of MICA and MICB genes after etoposide-induced stress**. We treated HaCaT and HeLa cells with 170 μM etoposide during 4 h. After RNA extraction and retrotranscription, Real-Time PCR was performed using GAPDH and β-Actin as reference genes. The "fold expression" (ordinates) was obtained by normalizing the data values with the untreated cells, which was arbitrarily set at 1. The figure represents the average ± SD of the relative expression obtained with the reference genes.

As we noted quantitative differences in the expression levels of MICA and MICB genes, we speculated that both genes might respond differentially to several stimuli, such as those induced by genotoxic stress. To address this question, one HPV-negative and one HPV-positive cell line (HaCaT and HeLa, respectively) were treated with 170 μM etoposide for 4 h and MICA and MICB mRNA abundance was assessed by Real-Time PCR using GAPDH and β-Actin as reference genes. After 4 h of treatment, we observed that genotoxic stress caused increase in MICA mRNA in HeLa cells (5.2-fold) and in HaCaT cells (68-fold) compared to untreated cells (Figure 6). Conversely, MICB mRNA increased only two-fold after etoposide treatment in both cell lines (Figure 6). While not as striking as the changes in MICA mRNA, levels of MICB mRNA increased over two-fold after etoposide treatment (Figure 6). To follow up on this result we also measured cell surface expression of MICA and MICB in HaCaT and HeLa cells after etoposide treatment, but no significant difference was observed (data not shown). A possible explanation for this discrepancy could be the short time frame of the experiment (4 h). However, the quantitative differences observed in the mRNA levels of MICA and MICB genes suggest that both genes might respond differentially to damage stimuli.

## Discussion

Since the discovery of "null" killer cells more than 30 years ago, there has been a large body of evidence implying the participation of NK cells in the recognition and lysis of tumor cells [37-39]. It is currently known that the activity of NK cells is delicately controlled by a balance between inhibitory and activating receptors [40]. NKG2D, one of the best characterized NK cell activating receptors, can promote tumor lysis upon recognition of MICA, MICB or the ULBPs. [41-43].

Up-regulated expression of MICA/B on the tumor cell surface has been considered a "danger signal" in order to activate NKG2D-expressing cells and promote anti-tumor immunity [4,13,42] However, numerous studies have demonstrated tumor evasion through metalloprotease-induced proteolytic release of MICA and MICB from the cell surface, which provokes down-regulation of NKG2D in NK and T cells [44-47]. Recently, we demonstrated increased soluble MICA levels in sera from patients with cervical cancer and precursor lesions as compared with healthy donors [48]. However, in that study we did not test a correlation between soluble MICA amounts and MICA expression in cervical tissues. Therefore, in the present study we performed a systematic analysis of the MICA and MICB expression in human cervical cancer cell lines. The majority of malignant cervical tumors are associated with infection by HPV-16 and HPV-18 [49]. Therefore, we chose SiHa and HeLa cells for our study, which are infected with these virus types. We also included an HPV-negative cervical cancer cell line (C33-A), as well as an HPV-negative, spontaneously immortalized human keratinocyte cell line (HaCaT) with a highly preserved differentiation capacity comparable to that of normal keratinocytes [35]. In this study we used different clones of antibodies against MICA/B (clone 6D4), MICA (clone 159227) and MICB (clone 236511). Interestingly, we observed starkly different MFI with the different antibodies. Of particular interest is the difference between the MICA/B MFI spread between SiHa and HeLa (189.54:116.71) that is not at all seen in the MICA staining of those cells, which were essentially identical (325.44:368.39). From this, we conclude that the common anti-MICA/B antibody must be binding different epitopes with different affinities than the individual antibodies. It is tempting to imagine that the individual MICA antibody binds an epitope that is conserved between HeLa and SiHa cells, while the MICA/B antibody binds an epitope that might be slightly different between the two cell lines, leading to different binding affinities. Our data on the preferential cell surface expression of MICA over MICB in SiHa cells are in agreement with a recent study examining activating NK ligands in various cell lines (including SiHa) and in tumor cells from patients with cervical cancer [50].

Due to the fact that the tumor cell lines tested in our study showed a significant cell surface expression of MIC molecules, the question is still open as to whether or not these tumor cells are able to escape from the normally efficient NK cell activation through the MIC/NKG2D pathway. For instance, sustained expression of NKG2D ligands has been shown to promote downregulation of the NK cell functions in transgenic mice constitutively expressing Rae-1ε [51]. One mechanism by which the persistent MIC ligand expression could affect NK cell activity is by shedding MIC molecules from the tumor cell surface. In other words, the constitutive MIC expression on the tumor cell surface might be a continuous source of soluble MIC molecules, which are thought to engage the NKG2D receptor and cause its internalization and subsequent lysosomal degradation [47]. The high levels of soluble MICB found in the present study suggest that this ligand might play a different biological role than MICA. In support of this is the fact that elevated soluble MICB correlated with disease activity in patients with multiple sclerosis during relapses while soluble MICA did not show any association with the disease; rather, the levels of this ligand were similar to healthy controls [52]. Therefore, an issue of vital importance will be to address if both soluble MICA and MICB share the same biological function in patients with HPV-associated tumors and to determine whether MICB, which was found in a higher concentration than MICA in the supernatants of cervical cancer cell lines, could promote NKG2D downmodulation in NK cells of cervical cancer patients. It will be also interesting to investigate if MICA and MICB show different mechanisms of posttranslational control; for instance, Agüera-González et al., showed recently that MICB has a short time of residence at the plasma membrane and demonstrated that MICB shedding was one of the mechanisms that contributes to the rapid loss of ligand from the cell surface [53]. Thus, if high levels of soluble MICB are present in the serum of cervical cancer patients, and this contributes to tumor immune escape, targeting of this molecule could be a promising alternative to improve tumor immunosurveillance in these patients.

The Real-Time RT-PCR results for MICB are especially interesting in light of the flow cytometry and ELISA data. While MICB transcripts were slightly lower than those for MICA in the HPV-positive cell lines, C33-A cells showed the opposite. The same finding could be observed by cell surface staining (with apparent MFIs of 84.60 *versus *24.67 and 40.06 for SiHa and HeLa, respectively) and also in the ELISA experiments for soluble MICB in the culture supernatant. Despite the fact that MICB appears to be less polymorphic than MICA, polymorphisms in the MICB promoter, with important variations in transcription rates, have been described [54]. If this is the case here, then these polymorphisms might partly explain the higher transcriptional rate that we observed in the C33-A cell line.

More importantly, the results with etoposide demonstrated that while MICB mRNA level rose slightly after treatment, MICA mRNA was strikingly upregulated, especially in non-tumorigenic HaCaT cells (68-fold increase). To follow up on this result, we also measured cell surface expression of MICA and MICB in HaCaT and HeLa cells after 4 h of etoposide treatment, but no significant difference was observed (data not shown). A possible explanation for this discrepancy might be the short time frame of the experiment. However, the quantitative differences observed in the mRNA levels of MICA and MICB genes in response to etoposide treatment suggest that both genes could be regulated in different ways. Indeed, a recent study that analyzed the architecture and function of the promoter of the MIC genes provided evidence for differential regulation of MICA and MICB [25].

Taken together, our results suggest that in spite of sharing a high degree of homology at both genomic and structural levels, MICA and MICB are differentially regulated at the transcriptional level and by cleavage at the cell surface in response to varying danger signals. Additionally, our data might point to roles for both ligands in the escape from immunosurveillance by tumor cells: sustained over-expression of MICA at the cell surface of HPV-positive cells, which could promote downregulation of the NK cell functions; and the shedding of MICB in HPV-negative cervical cancer cells, which could bind to NKG2D receptors and outcompete cell-surface activating ligands. Modulating the cell surface expression or targeting the proteases that mediate shedding of NKG2D ligands may open a new approach for the treatment of cervical cancer.

## Competing interests

The authors declare that they have no competing interests.

## Authors' contributions

STA conceived and designed the theoretical framework of the study, provided scientific guidance throughout the project and wrote the manuscript. NAG performed the experimental work, searched scientific literature and contributed to the draft of the manuscript. AAL provided scientific guidance throughout the project and participated in the experimental work. ACA and JH participated in the design of the study and contributed to the review of the manuscript. MJP, ATA, POL and GHF contributed with flow cytometry and Western blot experiments. PBN and OGR contributed to the draft of the manuscript and helped with editing. ABC and ADN contributed to the planning of the project and provided valuable scientific suggestions. LFJS conceived and designed the theoretical framework of the study, provided scientific guidance throughout the project and contributed to the writing of the manuscript. All authors helped to draft the manuscript and read and approved this final version.
